# A Case of Vasospastic Angina Possibly Triggered by Hyperthyroidism

**DOI:** 10.7759/cureus.96033

**Published:** 2025-11-03

**Authors:** Kazuki Suyama, Kazuki Haraguchi, Yoshiya Orita, Hisashi Koga, Tomohiro Kawasaki

**Affiliations:** 1 Medicine, Shin-Koga Hospital, Kurume, JPN; 2 Cardiology, Shin-Koga Hospital, Kurume, JPN

**Keywords:** coronary artery spasm, ergonovine stress test, graves' disease, hyperthyroidism, vasospastic angina

## Abstract

A 55-year-old man experienced chest tightness once a month. After half a year, he was admitted to our hospital for a thorough examination due to frequent chest symptoms and was diagnosed with vasospastic angina (VSA) following an ergonovine stress test on coronary angiography. The patient also had hyperthyroidism, which led to a diagnosis of Graves' disease. In this case, the patient did not exhibit typical hyperthyroid symptoms such as goiter, palpitations, excessive sweating, or finger tremors. However, the timing of weight loss coincided with an increase in the frequency of chest pain episodes, suggesting that hyperthyroidism may have contributed to the onset of VSA. Since hyperthyroidism has been reported to be a risk factor for VSA and its coexistence is known to increase the risk of severe coronary spasm, thyroid function should be evaluated aggressively in patients suspected of having VSA.

## Introduction

Vasospastic angina (VSA) is characterized by transient myocardial ischemia due to a temporary reduction in coronary blood flow caused by coronary artery spasm. Compared with atherosclerotic angina, which involves organic coronary artery stenosis, VSA generally has a favorable prognosis, with reported survival rates of 95%, 90%, and 87% at one, two, and three years, respectively [1]. However, recent reports have shown that even with appropriate pharmacological therapy, approximately 13% of patients experience recurrent anginal attacks, indicating that VSA can significantly affect the quality of life after onset [2]. Moreover, VSA can lead to serious complications such as myocardial infarction, life-threatening arrhythmias, or sudden cardiac death, highlighting the importance of accurate diagnosis and risk stratification [3].

On the other hand, hyperthyroidism is known to exert multifaceted effects on the cardiovascular system. It can increase cardiovascular risk through several mechanisms, including tachycardia, enhanced sympathetic nervous activity, elevated oxidative stress, and increased vascular reactivity [4]. Interestingly, although rare, several reports have described cases of VSA occurring in association with hyperthyroidism. Kim et al. reported that hyperthyroidism increased the risk of VSA by 3.27-fold [5]. One proposed mechanism is that, in hyperthyroidism, the excessive increase in reactive oxygen species (ROS) and dysregulation of the antioxidant system contribute to the development of cardiovascular dysfunction [4].

Nevertheless, reports of VSA secondary to hyperthyroidism remain extremely limited, and the underlying pathophysiological mechanisms, clinical characteristics of such cases, therapeutic strategies, and subsequent outcomes have not been fully elucidated. We experienced a case in which hyperthyroidism was considered a contributing factor in the development of VSA. This case provides valuable insights and may serve as a basis for further discussion and understanding of this rare association.

This article was previously presented as a meeting abstract at the 137th Kyushu Regional Meeting of the Japanese Circulation Society on December 14, 2024.

## Case presentation

A 55-year-old man had experienced episodes of chest tightness accompanied by a sensation of coldness once a month. The symptoms originated in the center of his chest and radiated to both sides of the neck and lower jaw, lasting for several minutes. These episodes occurred both at rest and during exertion but were most prominent from nighttime to early morning. Four months later, the frequency increased, so he visited the cardiology department of our hospital and underwent coronary CT, which showed mild coronary artery effects. Based on the symptoms, coronary VSA was suspected, and nitroglycerin sublingual tablets were prescribed. The symptoms persisted, but they improved within a few seconds with nitroglycerin as needed. Two months later, the patient experienced recurrent episodes lasting about one hour beginning at midnight and visited our outpatient cardiology clinic the next day, leading to hospitalization for further examination.

He had no notable past medical history. He had a 32-year smoking history (20-52 years of age, approximately 30 cigarettes per day) and consumed one to three 350mL cans of beer daily. His only medication was 0.3 mg sublingual nitroglycerin as needed. On admission, the patient was alert and oriented. His height was 160.5 cm and weight 59.7 kg, reflecting a four-kg weight loss over the preceding four months. Vital signs were as follows: temperature, 36.5°C; blood pressure, 123/76 mmHg; pulse rate, 92 beats per minute; respiratory rate, 19 breaths per minute; and oxygen saturation, 98% on room air. Physical examination, including cardiac, pulmonary, and abdominal findings, was unremarkable. Furthermore, classical signs of hyperthyroidism, including tremor, exophthalmos, sweating, and muscle weakness, were absent (Table 1).

**Table 1:** Physical findings on admission

Sysytem		Findings						
Neck		Thyroid not enlarged						
Face		No exophthalmos						
Heart sounds		No S1 or S2 accentuation, no S3 or S4, no heart murmurs						
Respiratory sounds		No rales						
Abdomen		No abnormalities noted						
Extremities		No edema, no tremor, no muscle weakness						
Skin		No coldness, no diaphoresis						

Laboratory data on admission showed no elevation of myocardial enzymes. Thyroid function testing demonstrated elevated free thyroxine (FT4, 3.589 ng/dL) and free triiodothyronine (FT3, 11.20 pg/mL), with suppressed thyroid-stimulating hormone (TSH <0.005 µIU/mL). TSH receptor antibody was elevated (3.1 IU/mL) (Table 2).

**Table 2:** Laboratory findings on admission WBC: White Blood Cell count; RBC: Red Blood Cell count; Hb: Hemoglobin; Ht: Hematocrit; Plt: Platelet count; TP: Total Protein; LDH: Lactate Dehydrogenase; CK: Creatine Kinase; CK-MB: Creatine Kinase-MB; TG: Triglyceride; HDL-C: High-Density Lipoprotein Cholesterol; LDL-C: Low-Density Lipoprotein Cholesterol; BUN: Blood Urea Nitrogen; Cre: Creatinine; AST: Aspartate Aminotransferase; ALT: Alanine Aminotransferase; Troponin I: Cardiac Troponin I; TSH: Thyroid-Stimulating Hormone; FT4: Free Thyroxine; FT3: Free Triiodothyronine; CRP: C-Reactive Protein; TgAb: Thyroglobulin Antibody; TPOAb: Thyroid Peroxidase Antibody; TRAb: TSH Receptor Antibody.

Parameter			Result		Reference range		Unit
WBC			5,2		3.5-9.0		×10^3^/µL
RBC			4.39		4.0-5.5		×10^6^/µL
Hb			6.90%		13.5-17.5		g/dL
Ht			38.5		39-52		%
Plt			346		150-350		×10^3^/µL
TP			6.9		6.5-8.0		g/dL
LDH			159		120-230		U/L
CK			49		60-240		U/L
CK-MB			0.8		<25		ng/mL
TG			109		30-149		mg/dL
HDL-C			39		40-90		mg/dL
LDL-C			61		70-139		mg/dL
BUN			15.2		8.0-20		mg/dL
Cre			0.52		0.65-1.07		mg/dL
AST			26		10-40		U/L
ALT			47		5-45		U/L
Troponin I			4		<0.04		pg/mL
TSH			<0.005		0.4-4.0		µIU/mL
FT4			3.58		0.9-1.7		ng/dL
FT3			11.2		2.3-4.0		pg/mL
CRP			0.2		<0.3		mg/dL
TgAb			16		<28		IU/L
TPOAb			12		<16		IU/L
TRAb			3.1		<2.0		IU/L

**Figure 1:**
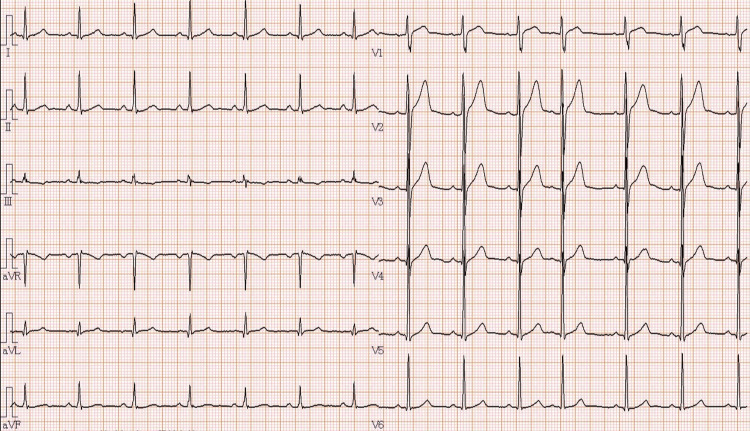
Resting electrocardiogram Heart rate, 81 beats per minute; sinus rhythm; no axis deviation; no ST-T changes; presence of premature atrial contractions (PACs)

Resting electrocardiography showed no ST-segment changes or arrhythmias (Figure 1).

**Figure 2:**
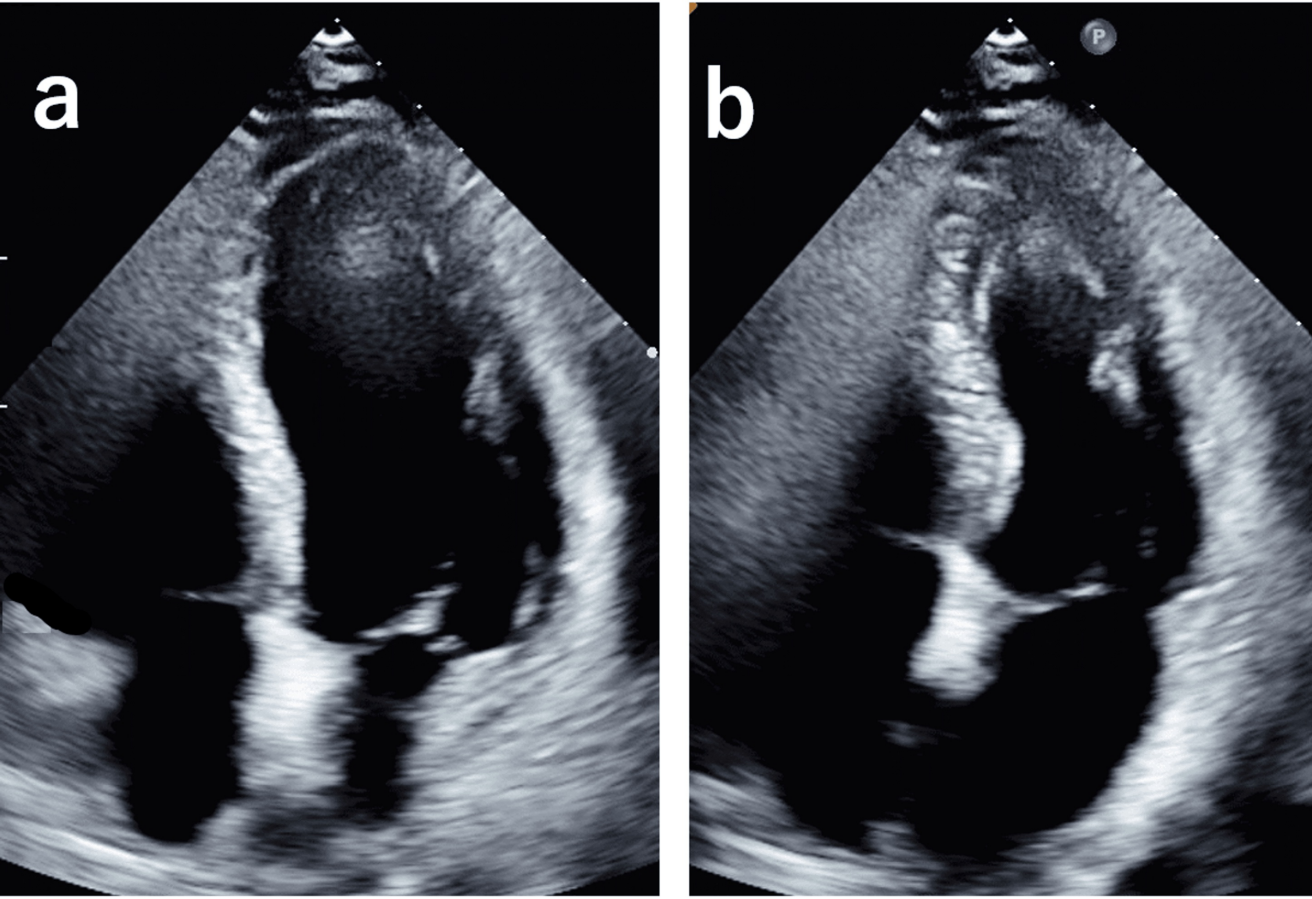
Echocardiography Apical four-chamber view: (a) diastole and (b) systole. Left ventricular ejection fraction: 58%; no significant valvular disease; no left ventricular wall motion abnormalities; no pericardial effusion

Transthoracic echocardiography showed no abnormalities, including wall motion defects (Figure 2).

**Figure 3:**
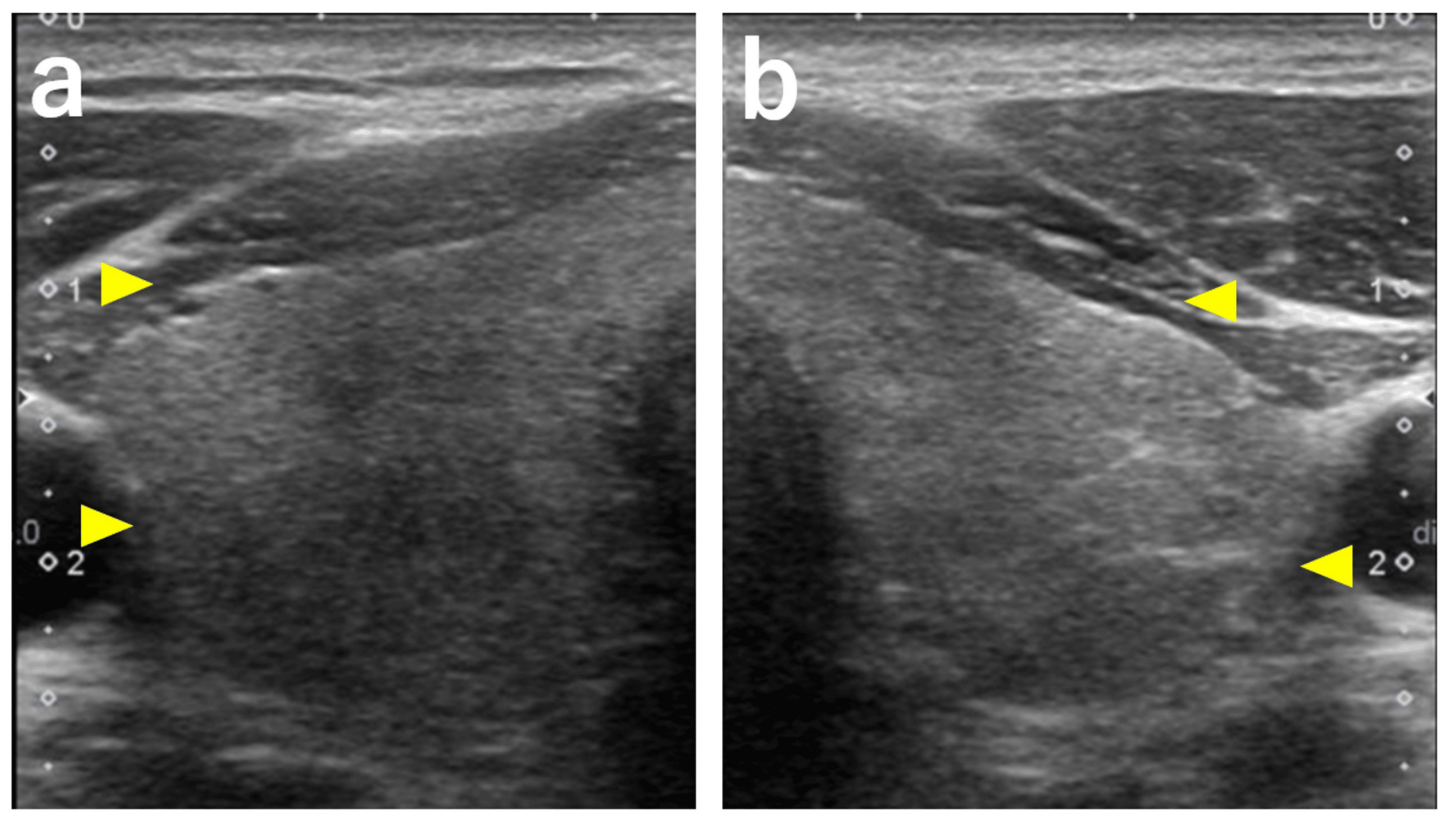
Thyroid ultrasonography (a) Right lobe and (b) Left lobe Mild thyroid enlargement with parenchymal coarseness, but no increased vascular flow was observed (arrowhead).

Thyroid ultrasonography revealed mild thyroid enlargement with heterogeneous echotexture, but no increased vascular flow (Figure 3).

**Figure 4:**
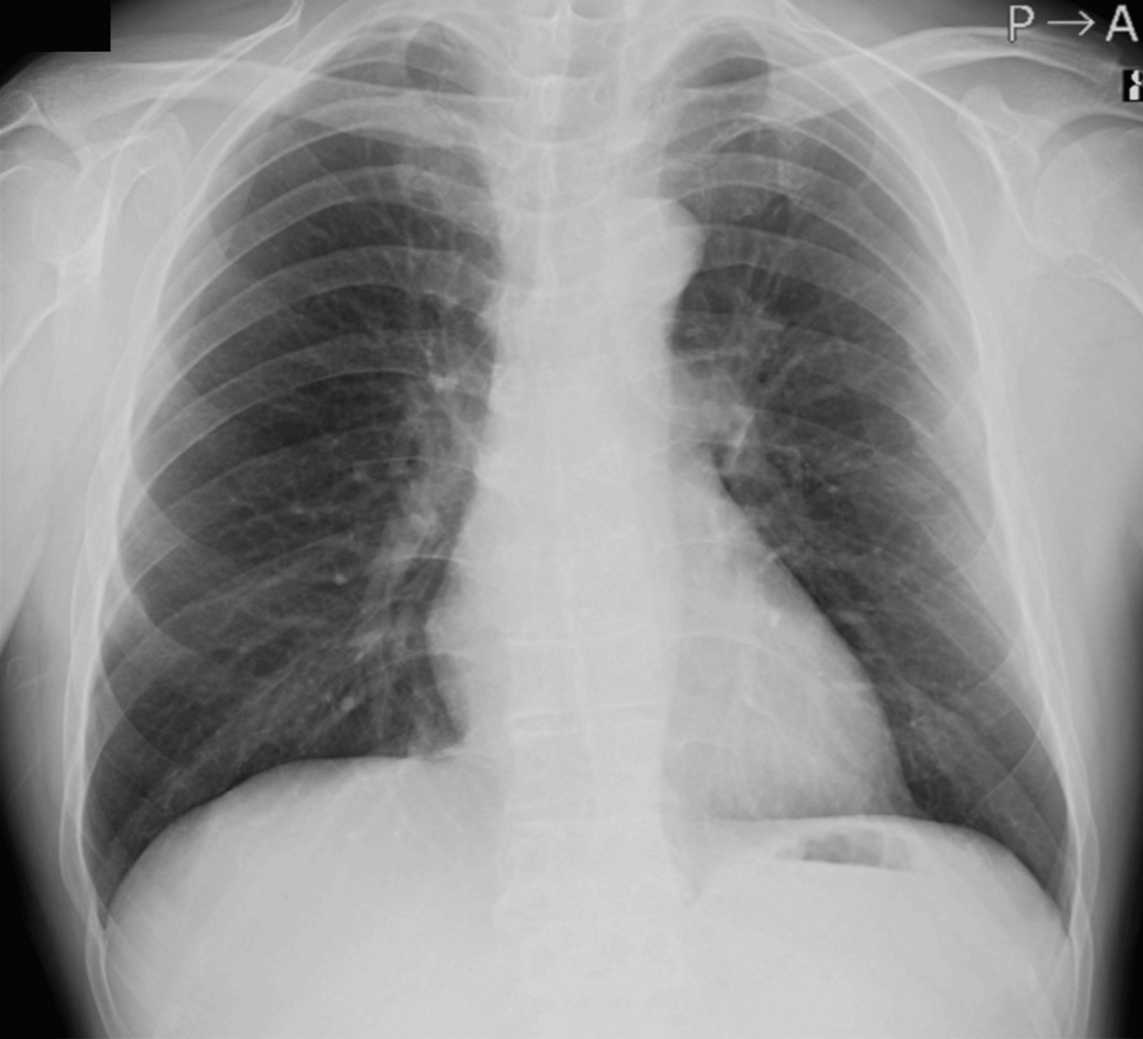
Chest radiograph No evidence of cardiomegaly, pleural effusion, or pulmonary congestion.

Chest radiography showed no cardiomegaly or pulmonary congestion (Figure 4).

**Figure 5:**
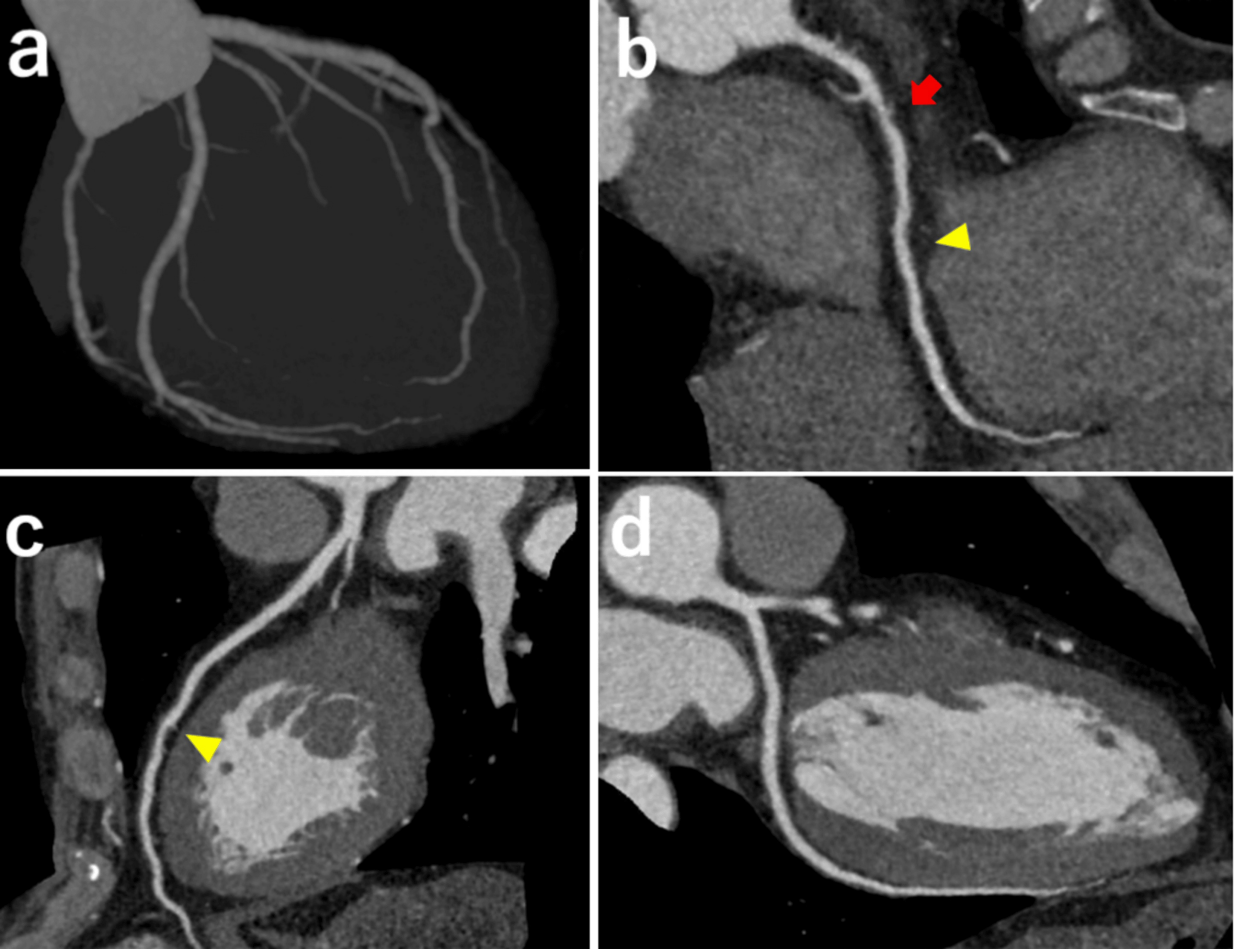
Coronary CT angiography (a) Overall view, (b) Right coronary artery — moderate stenosis in segment #1 (arrow) and mild stenosis in #2 (arrowhead), (c) Left anterior descending artery — mild stenosis in #7 (arrowhead), and (d) Left circumflex artery — no remarkable stenosis

Coronary computed tomography demonstrated moderate stenosis in segment #1 and mild stenosis in segment #2 of the right coronary artery and #7 of the left anterior descending artery (Figure 5).

Hospital course

**Figure 6:**
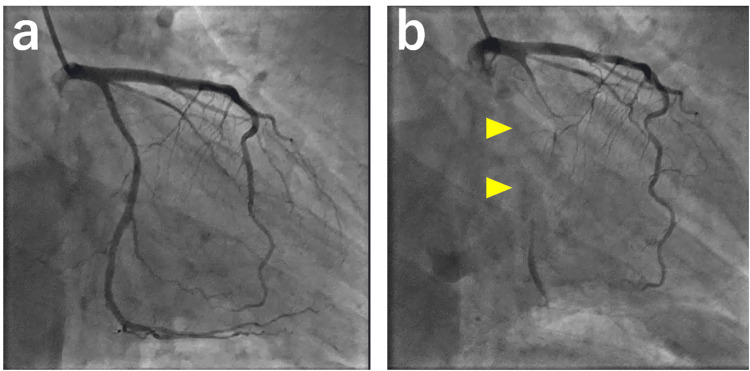
Coronary angiography and ergonovine stress test Angiography of the left coronary artery (RAO-caudal view). Ergonovine was administered at doses of 16 µg into the right coronary artery and 32 µg into the left coronary artery. (a) Control angiography (b) Angiography after ergonovine administration, showing complete occlusion of the left circumflex artery (arrowhead).

On the first day of admission, cardiac catheterization with an ergonovine provocation test was performed, revealing complete occlusion of the left circumflex artery (Figure 6).

**Figure 7:**
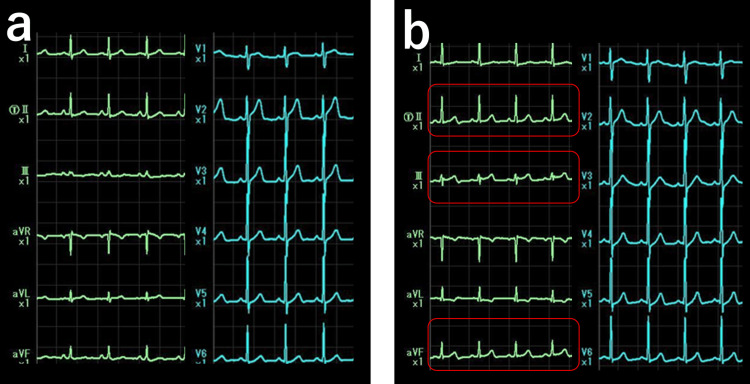
Electrocardiogram before and after ergonovine provocation test (a) Controle electrocardiogram. (b) ST elevation is observed in leads II, III, and aVF after ergonovine administration.

Concurrently, electrocardiogram showed ST-segment elevation in leads II, III, and aVF (Figure 7), and the patient experienced recurrence of his usual chest discomfort. The symptoms resolved after administration of isosorbide dinitrate. Since coronary angiography showed no significant stenosis, a diagnosis of VSA was confirmed, and treatment with diltiazem (200 mg/day) was initiated.

Meanwhile, the patient had noticed a weight loss of 4 kg over the past four months. Although classic hyperthyroid symptoms such as excessive sweating, finger tremors, palpitations, and exophthalmos were absent, laboratory data at admission revealed hyperthyroidism with decreased TSH and elevated FT3 and FT4 levels. Moreover, TSH receptor antibody levels were elevated, and thyroid ultrasound confirmed thyroid enlargement. Based on these findings, the patient was diagnosed with Graves' disease, and thiamazole (15 mg/day) was initiated on the third hospital day.

A 24-hour Holter ECG on the fourth day showed no significant arrhythmias or ST changes, and there was no recurrence of chest symptoms (Table 3), so the patient was discharged on the fifth hospital day.

**Table 3:** Holter electrocardiogram (ECG) findings

Parameter				Findings	
Recording duration				24 hours	
Total heartbeats				126.800 beats	
Mean heart rate				84 bpm	
Minimum heart rate				68 bpm	
Maximum heart rate				114 bpm	
Premature atrial contractions				558 beats	
Premature ventricular contractions				1 beats	
Supraventricular tachycardia				None	
Ventricular tachycardia				None	
ST-segment changes				None	
Sinus pause				None	
Atrial fibrillation				None	
Symptom				None	

Follow-up

At two months after discharge, TSH and FT4 levels had normalized, resulting in a reduction of methimazole to 10 mg/day. Thereafter, no recurrence of symptoms was observed at four months following discharge.

## Discussion

Atrial fibrillation is a well-recognized cardiac complication of hyperthyroidism; however, VSA has also been occasionally reported in association with hyperthyroidism, with a reported prevalence of 7.4% [6]. In a prospective study of 1,239 patients with suspected VSA, Kim et al. demonstrated that hyperthyroidism was associated with a 3.27-fold increase in the relative risk of developing VSA, which is greater than the relative risks conferred by established risk factors such as smoking (1.92-fold) and male sex (2.19-fold) [5].

The precise mechanism linking hyperthyroidism and VSA remains unclear. Hyperthyroidism is known to enhance sympathetic activity by increasing both the number and sensitivity of adrenergic receptors [7]. Conversely, VSA is thought to result from an exaggerated sympathetic response following transient parasympathetic activation [8]. These mechanisms suggest a plausible pathophysiological connection between the two conditions. 

In addition, a deficiency in endothelial nitric oxide (NO) activity has been reported in spastic coronary arteries, leading to supersensitivity to the vasodilatory effect of nitroglycerin and to the vasoconstrictive effect of acetylcholine in patients with VSA. This impaired endothelial NO activity plays an important role in the pathogenesis of coronary spasm [9]. Thyroid hormones, on the other hand, induce greater generation of NO in both the aorta and vascular smooth muscle cells [10]. At first glance, this appears to contradict the occurrence of coronary spasm in hyperthyroidism. However, under conditions of excessive NO production, superoxide (O₂∙⁻) can react with NO to form the highly reactive oxidant peroxynitrite (ONOO⁻), which not only inactivates NO but may also induce endothelial dysfunction through oxidative stress. Thus, this mechanism may contribute to the induction of coronary spasm in hyperthyroidism [4].

In the present case, coronary CT revealed no significant coronary stenosis, and based on findings from echocardiography, electrocardiography, and cardiac enzyme levels, acute coronary syndrome, myocarditis, and takotsubo cardiomyopathy were considered unlikely. The patient was ultimately diagnosed with VSA based on a positive ergonovine provocation test. Furthermore, this patient did not exhibit classic hyperthyroid symptoms, such as goiter, palpitations, excessive sweating, or finger tremors. However, the onset of weight loss coincided with a marked increase in the frequency of chest pain, implying that hyperthyroidism may have contributed to the development of VSA.

Previous case reports have similarly noted that many patients with hyperthyroidism-associated VSA lacked typical symptoms and that the diagnosis was often made incidentally through the thyroid function test performed during routine evaluation on admission [11,12]. Therefore, hyperthyroidism complicated by VSA may manifest only subtle or nonspecific clinical findings.

In the present case, no classic symptoms were observed. Thyroid enlargement was detectable only by ultrasound and was not apparent on physical examination. The patient experienced a four-kg weight loss over four months, which paralleled the exacerbation of chest symptoms. This temporal association suggests that weight loss in patients with VSA should raise suspicion for underlying hyperthyroidism, even if minimal at symptom onset. Furthermore, although approximately 80% of patients with hyperthyroidism (Graves' disease) are women [13], this patient was male, underscoring the importance of thyroid function testing in all VSA patients, regardless of sex or the presence of overt hyperthyroidism features.

Previous studies have also reported that patients with VSA associated with hyperthyroidism present more frequently with acute myocardial infarction, diffuse-type spasm, left main coronary involvement, and medically refractory vasospasm compared with those without thyroid dysfunction [6]. These findings suggest that hyperthyroidism-related VSA may exhibit more severe clinical manifestation. Moreover, adverse cardiovascular events are reportedly more common in hyperthyroid patients who develop VSA [5]. Accordingly, careful monitoring for coronary spasm should be considered during the follow-up of patients with hyperthyroidism.

Conversely, when appropriate treatment for hyperthyroidism is initiated, the long-term prognosis of patients with VSA does not differ significantly from that of patients without thyroid dysfunction [6]. There are also reports describing successful early discontinuation of calcium channel blockers without recurrence of anginal symptoms following normalization of thyroid function [14]. In the present case, treatment for hyperthyroidism was initiated concurrently with standard VSA therapy, resulting in normalization of thyroid function and complete resolution of chest symptoms during a four-month follow-up after discharge. These findings support the concept that optimal control of thyroid function is integral to the effective management of VSA.

## Conclusions

This case supports the hypothesis of a causal relationship between VSA and hyperthyroidism. It suggests that in patients presenting with marked weight loss, the possibility of coexisting hyperthyroidism should be considered, and that even subclinical or atypical hyperthyroidism may contribute to the development of VSA. Therefore, it is advisable to perform early screening for hyperthyroidism in patients with VSA regardless of age or sex. Since treatment of hyperthyroidism can lead to improvement of anginal symptoms, integrated management tailored to each case is essential. The accumulation of similar cases in the future is expected to contribute to better risk assessment and optimization of therapeutic strategies for VSA.
